# What May Constrain the Success of Indoleamine 2,3-Dioxygenase 1 Inhibitors in Cancer Immunotherapy?

**DOI:** 10.3389/fimmu.2018.01879

**Published:** 2018-08-13

**Authors:** Theodoros Eleftheriadis

**Affiliations:** Department of Nephrology, Faculty of Medicine, University of Thessaly, Larissa, Greece

**Keywords:** indoleamine 2,3-dioxygenase, cancer immunotherapy, p53, metabolism, T-cell

Indoleamine 2,3-dioxygenase 1 (IDO1) catalyzes the initial rate-limiting step of tryptophan degradation along the kynurenine pathway and suppresses T-cell immune response by two paths; the activation of general control non-derepressible 2 kinase (GCN2K) and aryl-hydrocarbon receptor (AhR). In the microenvironment of the immune response, tryptophan depletion activates GCN2K, which inhibits T-cell proliferation and induces T-cell apoptosis (1). From a teleological point of view, the selection of tryptophan depletion as an immunomodulatory mechanism is ingenious. Tryptophan is an essential amino acid not synthesized by human cells, its concentration in the body is the lowest among all amino acids, and its deprivation due to low intake appears only in 2 days (2). Thus, its depletion in the microenvironment of inflammation can emerge acutely. Interestingly, and indicating the specific role of the above immunomodulatory mechanism, IDO1-induced tryptophan depletion does not affect the other amino acid sensing system, the mammalian target of rapamycin complex 1 (mTORC1), in T-cells (3–5), which is in accordance with studies showing that mTORC1 is sensitive to the depletion of specific amino acids; more precisely of leucine, isoleucine, valine, and possibly arginine, but not of tryptophan (6). In parallel with IDO1-induced GCN2K activation, kynurenine, a derivative of tryptophan degradation, activates AhR, which induces naïve CD4+ T-cell differentiation into regulatory T-cells (7).

The immunosuppressive properties of IDO1 were discovered by the observation that its expression in the placenta contributes to a successful semi-allogenic pregnancy (8). Then it was revealed that inflammatory stimuli induce IDO1 expression in antigen-presenting cells, and the immunosuppressive role of this enzyme has been confirmed in experimental models of autoimmunity and transplantation (9–12).

Indoleamine 2,3-dioxygenase 1 is also expressed in many types of cancer, and the majority of studies suggest that this enzyme plays a significant role in the escape of tumors from immunosurveillance (13, 14). More precisely in various types of cancer, IDO1 expression has been confirmed, individually or in combination, in tumor cells, in interstitial cells in lymphocyte-rich areas, and in endothelial cells. In most cases, IDO1 expression seems to be the result of an ongoing immune response by infiltrating T-cells and other immune cells that produce interferon-γ (IFN-γ) (14, 15), a cytokine that induces macrophage and dendritic cell (DC) activation and IDO1 expression (13, 14, 16). The infiltrating immune cells fail to eliminate cancer cells because due to accumulated mutations they escape the initial immune response. The persisted immune response results in increased IDO1 expression by tolerogenic DCs, myeloid-derived suppressor cells, and tumor-associated macrophages. Tryptophan depletion and kynurenine production by IDO1 induce more immune cells to become tolerogenic and inhibit effector T-cells, whereas increase regulatory T-cells. Regulatory T-cells by expressing cytotoxic T-lymphocyte-associated-antigen-4 (CTLA-4) inhibit further effector T-cells and increase IDO1 expression in DCs closing a positive feedback loop of immunosuppression (16). However, in a subset of tumors IDO1 is expressed by cancer cells in the absence of any inflammation indicating that it may be the result of oncogenic events and may contribute to escape of tumor by immunosurveillance by preventing T-cell infiltration (14, 15).

Nevertheless, other studies question the role of IDO1 overexpression in the adverse clinical outcome of certain cancers. Ishio et al. found that the recurrence-free survival rate of patients with IDO1-positive hepatocellular carcinoma is significantly higher than that of patients with IDO1-negative hepatocellular carcinoma (17). Takao et al. showed that increased IDO1 protein is related to worse prognosis in patients with serous type, but not with clear cell or endometrioid type of ovarian adenocarcinoma (18). Riesenberg et al. revealed that the expression of IDO1 in tumor endothelial cells correlates with long-term survival of patients with renal cell carcinoma (19). Jacquemier et al. determined that high IDO expression is associated with morphological medullary features and has an independent favorable prognostic value in patients with basal-like breast carcinoma (20). Recently, Heeren et al. showed that in patients with early stage cervical cancer, a marginal IDO expression pattern in the tumor dominantly predicts a favorable outcome, which might be related to IFN-γ release in the cervical tumor microenvironment (21).

Most importantly, despite the initial experimental and clinical indications about the efficacy of IDO1 inhibitors in cancer immunotherapy (16), in the recently Incyte’s phase III clinical trial, the addition of the IDO1 inhibitor epacadostat in a therapy with the programmed death 1 immune checkpoint inhibitor pembrolizumab, made no difference for the patients with metastatic melanoma receiving both drugs. This failure led three companies to the decision to suspend, cancel, or downsize 13 trials of IDO1 inhibitors in combination with immune checkpoint inhibitors (22).

There are some possible explanations for these disappointing results. First, IDO1 expression, confirmed by either immunohistochemistry or polymerase chain reaction, in a tumor does not necessarily mean that this enzyme is functional. For instance, IFN-γ induces both the expression of IDO1 and the production of nitrogen monoxide (NO) in macrophages, but the latter inhibits IDO1 enzymatic activity (23). Also, in an inflammatory environment, both NO and superoxide anion are produced resulting in the generation of peroxynitrite anion, which inhibits by nitration IDO1 enzymatic activity without affecting its protein level (24). Moreover, phosphorylation of specific IDO1 tyrosine residues blocks its catalytic activity (25). Thus, assessing along with IDO1 expression its enzymatic activity by detecting in the tumors along with IDO1, proteins that are known to be modified or expressed after GCN2K or AhR activation would yield more accurate results about the role of this enzyme in the escape of tumors from immunosurveillance.

In addition, IDO1, by activating GCN2K, alters the metabolism of T-cells, inhibits their proliferation and induces apoptosis in a p53-dependent way (4, 26, 27). The transcription factor p53, also known as tumor suppressor p53, inhibits aerobic glycolysis, which characterizes rapidly proliferating cells, and induces cell cycle arrest and/or apoptosis (28, 29). Interestingly, activated GCN2K also increases p53 expression in nonimmune cells, such as human aortic endothelial and renal epithelial cells (30, 31). The fact that in most the tumors the p53 pathway is directly or indirectly inactivated (28), offers an advantage in cancer progression. IDO1 expressed by cancer cells or infiltrating immune cells by depleting tryptophan in the local microenvironment activates GCN2K in the T-cells that infiltrate the lesion inhibiting their proliferation and inducing apoptosis. On the contrary, due to the ineffective p53 pathway in the cancer cells, tryptophan depletion does not inhibit tumor growth. Acting in such a way, IDO1 contributes to the escape of cancer from the immunosurveillance. However, in the case of cancer with the intact p53 pathway, the IDO1 expressed by the infiltrating immune cells may be able to activate GCN2K in cancer cells and inhibit tumor progression in a p53-dependent way. In such a case, the administration of an IDO1 inhibitor may decrease the antitumor immune response. Interestingly, in an experimental study, IFN-γ exhibited its antiproliferative effects only in cancer cell lines in which it upregulated IDO1 expression with a consequent tryptophan deprivation; suggesting a possible direct antitumor effect of this enzyme in certain types of cancer. However, the p53 pathway was not assessed in the tested cancer cell lines (32). Thus, evaluation of the cancer p53 status before the administration of an IDO1 inhibitor may be vital.

Also, and despite the studies about the role of IDO1 in supporting tumor vessel formation (33, 34), the ability of activated GCN2K to induce p53 expression, and possibly cell cycle arrest or apoptosis, in endothelial cells (30), raises questions about the effect of IDO1 inhibition on the required for the tumor progression neoangiogenesis. Interestingly, expression of IDO1 in endothelial cells of renal tumors is associated with a better prognosis (19).

As regards the immunosuppressive properties of IDO1 *per se*, research in my laboratory, revealed that this enzyme affects T-cell fate at least in part by altering cell metabolism (3–5, 26, 35, 36). Thus, the availability of various nutrients in the microenvironment of the immune response may have a significant impact on IDO1 immunomodulatory properties. Most of the conclusions about the molecular pathways involved in the IDO1-induced immunosuppression were extrapolated under the strictly controlled conditions of cell cultures (1, 7). Nevertheless, if a free fatty acid is added in the culture medium, the trend for CD4+ T-cell differentiation toward a regulatory phenotype remains, but the antiproliferative and pro-apoptotic properties of IDO1 disappear (35, 36). The reason relies on the effect of IDO1 on T-cell metabolism. As depicted in more detail in Figure 1, depletion of tryptophan by activating GCN2K inhibits glucose and glutamine catabolism (3, 4, 26, 36). However, kynurenine by activating AhR induces free fatty acid β-oxidation, which refuels CD4+ T-cells with energy, allowing their proliferation and preventing their apoptosis (35, 36). Accordingly, two of the three ways by which IDO1 is supposed to suppress T-cell-mediated immune response may not take place if enough free fatty acids are present in the cancer microenvironment. In such a case, the gain in antitumor immunity by inhibition of IDO1 would be far less than the expected. The data about the concentration of free fatty acids in the various types of cancer are scarce.

**Figure 1:**
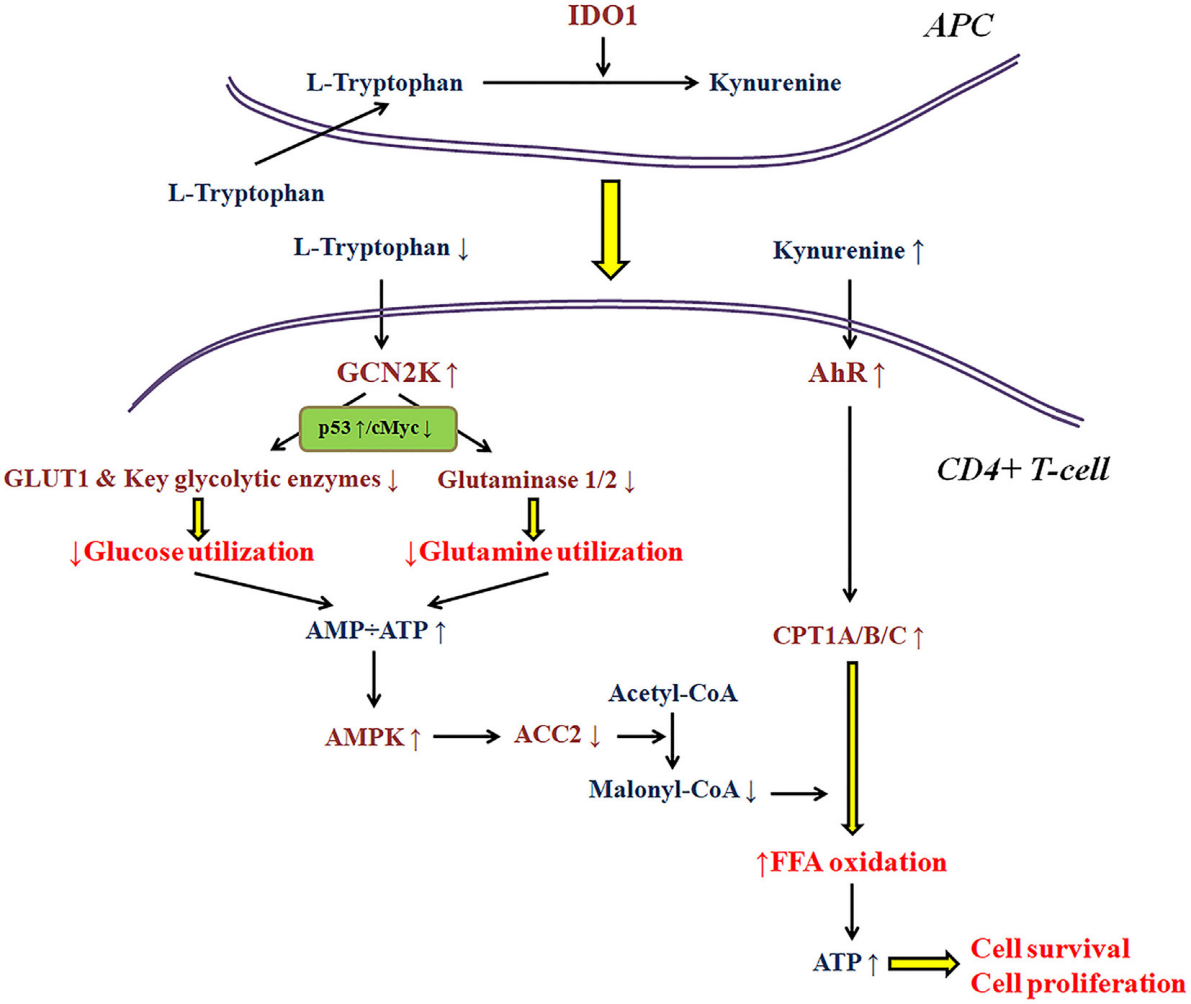
A model about the effect of indoleamine 2,3-dioxygenase 1 (IDO1) on the utilization of the main energy sources by activated CD4+ T-cells. In the immune response microenvironment, IDO1 by degrading l-tryptophan along the kynurenine pathway activates general control non-derepressible 2 kinase (GCN2K) and aryl-hydrocarbon receptor (AhR). By upregulating the transcription factor p53 and downregulating the transcription factor c-Myc, activated GCN2K decreases the expression of glucose transporter 1 (GLUT1), key glycolytic enzymes, and glutaminases inhibiting the consumption of glucose and glutamine. The reduced utilization of these pivotal sources of energy by activated T-cells results in reduced ATP production. The latter activates AMP-activated protein kinase (AMPK), which phosphorylates and inactivates acetyl-CoA carboxylase 2 (ACC2) resulting in decreased production of the carnitine palmitoyltransferase I (CPT1) inhibitor malonyl-CoA. In parallel, activation of AhR increases the expression of all CPT1 isoenzymes. Since CPT1 controls free fatty acid oxidation, these IDO-induced alterations promote free fatty acid oxidation as an alternative fuel for ATP production, supplying the required energy for CD4+ T-cell survival and proliferation.

In conclusion, there are many aspects to be revealed about the role of IDO1 in the escape of cancer from immunosurveillance (Table 1). Along with tumor IDO1 expression, assessment of its activity may prevent overestimation of its role in the escape of cancer from immunosurveillance. In cancer with an intact p53 pathway, expression of IDO1 by the infiltrating immune cells may exhibit antitumor activity. Also, in an environment relatively rich in free fatty acids the immunosuppressive properties of IDO1 may be decreased considerably, and the gain in antitumor immunity from its inhibition may be less than the expected. The role of IDO1 in tumor neoangiogenesis remains to be better elucidated as well. Administration of IDO1 inhibitors may be beneficial to certain but not all cancers. Beyond tumor IDO1 expression, assessment of other factors such as IDO enzymatic activity, the status of the p53 pathway in the cancer cells, and the availability of free fatty acids in the tumor microenvironment, i.e., the application of a more personalized medicine, may help IDO1 inhibitors to find their place in cancer immunotherapy.

**Table 1:** Factors that may limit the anticancer effect of indoleamine 2,3-dioxygenase 1 (IDO1) inhibitors.

The role of IDO1 in the escape of cancer from immunosurveillance may be overestimated	Most studies assessed only IDO1 expression but not its activity. However, certain conditions that may be present in the cancer microenvironment may inhibit IDO1 activity without affecting its protein level
In certain tumors, IDO1 may induce apoptosis of the cancer cells	In human lymphocytes, epithelial and endothelial cells, IDO1 by activating general control non-derepressible 2 kinase (GCN2K) induces p53-mediated apoptosis. Thus, in the minority of cancers with an intact p53 pathway, IDO1 expression in the infiltrating immune cells may be beneficial
In certain tumors, IDO1 may suppress neoangiogenesis	Although there are studies that support a positive role for IDO1 in tumor neoangiogenesis, the fact that GCN2K activation induces p53-mediated apoptosis in human endothelial cells raises questions. In renal carcinoma, the expression of IDO1 in endothelial cells signifies a worse prognosis
The immunosuppressive properties of IDO1 may be overestimated	Traditionally, it is thought that IDO1 suppresses T-cells proliferation, induces their apoptosis, and promotes their differentiation toward a regulatory phenotype. However, the presence of free fatty acids in the tumor microenvironment may abolish the antiproliferative and pro-apoptotic properties of IDO1

## Author Contributions

The author confirms being the sole contributor of this work and approved it for publication.

## Conflict of Interest Statement

The author declares that the research was conducted in the absence of any commercial or financial relationships that could be construed as a potential conflict of interest.
